# A Comparative Study of the Role of Formins in *Drosophila* Embryonic Dorsal Closure

**DOI:** 10.3390/cells11091539

**Published:** 2022-05-04

**Authors:** Krisztina Tóth, István Földi, József Mihály

**Affiliations:** 1 Biological Research Centre, Institute of Genetics, Temesvári krt. 62, H-6726 Szeged, Hungary; toth.krisztina@brc.hu (K.T.); foldi.istvan@brc.hu (I.F.); 2 Doctoral School of Multidisciplinary Medical Science, Faculty of Medicine, University of Szeged, H-6725 Szeged, Hungary; 3 Department of Genetics, University of Szeged, H-6726 Szeged, Hungary

**Keywords:** dorsal closure, actin, formin, *Drosophila*

## Abstract

Dorsal closure is a late embryogenesis process required to seal the epidermal hole on the dorsal side of the *Drosophila* embryo. This process involves the coordination of several forces generated in the epidermal cell layer and in the amnioserosa cells, covering the hole. Ultimately, these forces arise due to cytoskeletal rearrangements that induce changes in cell shape and result in tissue movement. While a number of cytoskeleton regulatory proteins have already been linked to dorsal closure, here we expand this list by demonstrating that four of the six *Drosophila* formin type actin assembly factors are needed to bring about the proper fusion of the epithelia. An analysis of the morphological and dynamic properties of dorsal closure in formin mutants revealed a differential contribution for each formin, although we found evidence for functional redundancies as well. Therefore, we propose that the four formins promote the formation of several, and only partly identical, actin structures each with a specific role in the mechanics of dorsal closure.

## 1. Introduction

The establishment of tissue integrity during development or after injury often requires the fusion of two epithelial sheets. *Drosophila* embryonic dorsal closure (DC) is a broadly used model system to study the in vivo mechanisms of such closure processes. DC is a late morphogenetic process occurring after germ band retraction that leaves a hole on the dorsal side of the embryo, covered by the extraembryonic amnioserosa (AS) cells (reviewed in [1]). The closure of the epidermal gap is achieved by the migration of the two lateral epidermal cell sheets towards the dorsal midline, where they meet and fuse together while the AS cells contract and ultimately die by apoptosis. The initiation of DC begins at stage 12 when cells of the AS start to reduce the size of their apical surface through a series of rhythmic contractions, lasting until the completion of DC [2]. At stage 13, during the second phase of the process, the dorsal-most epidermal cells (DME) elongate in a dorsoventral direction, and they begin to produce a contractile, supracellular actin cable around the dorsal gap [2]. Subsequently, at stage 14, the two opposite epithelial sheets get close enough at the canthi of the hole to contact each other by filopodia and lamellipodia protruding from the DME or leading edge (LE) cells. These cellular protrusions help the zipping of the epidermal cell sheets and promote the formation of a seamless contact along the midline by stage 15 of embryogenesis [2].

**Figure 1:**
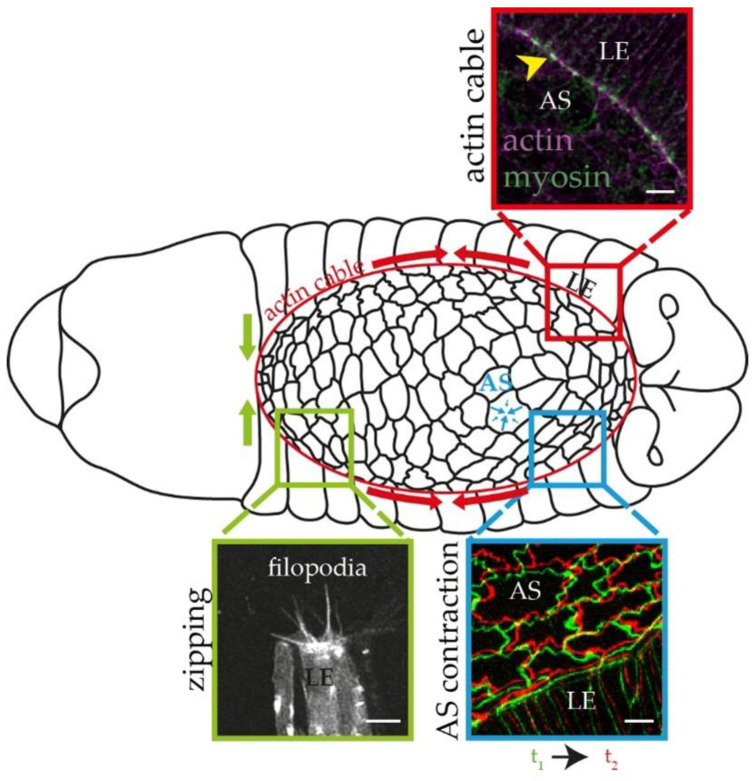
Schematic drawing of the process of dorsal closure in *Drosophila* embryos. Red arrows indicate the contraction of the actin cable around the dorsal gap. Red magnified region: a *w^1118^* embryo stained for actin (in magenta) and non-muscle Myosin II (in green) in the LE and the AS cells. Note that the actin and myosin stainings co-localize at the actin cable (arrowhead). Green arrows indicate the zipping forces at the LE cells. Green magnified region: LE cells from a single hemisegment of an *en-Gal4, UAS-Moe::mCherry* embryo, exhibiting filopodia. Blue arrows indicate the contraction of the AS cells. Blue magnified region: AS cells from an Arm::GFP-expressing embryo, indicating the displacement of the cell boundaries at two timepoints (t1 and t2) of the contraction. Scale bars: 5 μm.

Perfect sealing of the dorsal epidermis requires the concerted action of several forces. These include the apical contraction of the AS cells, exerting a pulling force on the lateral epidermis; the contraction of the actin cable flanking the dorsal hole; and the cell adhesion forces generated during the zippering process (Figure 1). While it has been shown that the loss of one of these forces can be partially compensated for by the others [3,4,5,6], it was also demonstrated that the contraction of the AS cells is the most important force-generating component during DC [5,6]. The closing process is based on dynamic cell shape changes driven by cytoskeletal rearrangements [7]. Zipping involves the coordinated action of the actin and microtubule cytoskeleton [2,8], while the contraction around the dorsal gap and that of the AS cells are generated by actomyosin cables. Research over the last few decades has identified a plethora of proteins that contribute to the formation and regulation of these actomyosin systems. For example, the JNK and Dpp signaling pathways [9,10,11] promote the formation of the supracellular actomyosin cable around the dorsal gap. The Z band alternatively spliced PDZ-motif protein 52 (ZASP52) is required to build these actin cables [6], the contractility of which is regulated by Rho GTPases [2]. In addition, it was shown that the heavy and light chain components of non-muscle Myosin II are essential for DC [12,13], and two formin types of actin assembly factors, Dia and Frl, also play a role [14,15].

Members of the highly conserved formin protein family belong to the major cytoskeleton regulators as they act as de novo actin nucleation factors and also support filament elongation (reviewed in [16]). Beyond actin regulation, many formins have been shown to interact with microtubules, and they play a role in crosslinking of the actin and microtubule cytoskeleton [17,18,19,20,21,22,23]. The genomes of the vertebrate species encode 15 formins [24], whereas the *Drosophila* genome encodes 6 formins, Dia (diaphanous), Frl (formin-related in leukocytes/formin-like), DAAM (disheveled associated activator of morphogenesis), Fhos (formin homology 2 domain-containing), Capu (cappuccino), and Form3 (Formin 3). Of these, Dia, thought to be indispensable for cytokinesis [25,26,27], is implicated in multiple aspects of DC, such as the regulation of actomyosin contraction in LE cells [14] and filopodia formation in both LE and AS cells [28,29]. Frl is the other formin linked to DC by promoting the assembly of a medioapical actin subpopulation in AS cells [15]. Interestingly, a LOF analysis of these formins revealed that neither of them are essential for DC, rather they affect the cellular dynamics of the process. These findings highlight the robustness of the dorsal closure process and indicate that various actin regulators might have a specific contribution by supporting specific actin subpopulations. Thus, it remained possible that additional formin types of actin regulators were also involved, possibly impacting different actin networks than those controlled by Dia or Frl. To address this question, we decided to perform a comprehensive analysis of the six *Drosophila* formins during DC. Here, we present these studies revealing that, besides Dia and Frl, Form3 and DAAM are also expressed in LE and AS cells and play a role in the proper sealing of the dorsal epidermis by differentially regulating the underlying actin-dependent forces.

## 2. Materials and Methods

### 2.1. Drosophila Stocks and Genetics

Flies were raised at 25 °C under standard conditions. The following mutant strains were used: *w^1118^* (BL *#3605*), *Form3-GFSTF* (BL #65385), *Capu-GFSTF* (BL #66507), *Arm::GFP* (BL #8556), and *69B-Gal4* (BL #1774) provided by the Bloomington Drosophila Stock Center, *dDAAM^Ex4^* [30], *en-Gal4,UAS-Moe::mCherry* [31], *frl^59^* [15]; *dia^1^/CyO* [32], and *form3^1^* (see below). Where necessary, zygotic mutants were selected by using a *CyO, twi-Gal4, UAS-EGFP,* or *TM3*, *twi-Gal4, UAS-EGFP* balancer chromosome; protein and mRNA expression data were retrieved through FlyBase, the Drosophila databank [33]. The *UAS-FL-Form3* transgenic line was generated with standard cloning techniques by using the pTWF-attB vector.

The *form3^1^* mutant was generated by the CRISPR/Cas9 technique. Two 20 bp long gRNAs (TCGCCACCTGTCCTCCGGA and TGGGTCGCATGAAGCTGCT) were designed with homology to the first intron and to the last coding exon of *form3,* before cloning into the pCFD4 vector. To facilitate the identification of the expected deletions, we used an insertional mutant that carried a GFP marker in the gene (BL # 23411). After co-injection of the guide RNA expressing plasmid with Cas9 into this stock, we selected for the loss of the GFP marker in the larval progeny, and candidates picked up this way were subsequently validated by PCR and sequencing. Based on the sequencing data, the expected 13,559 bp deletion was detected in the genomic DNA of the mutant strain. In this stock, only 37 bps remained from the coding region, and thus we consider *form3^1^* as a protein null allele, which is homozygous viable and fertile, although it exhibits reduced viability and fertility when compared to the wild type.

### 2.2. Antibody Generation

The Frl antibody was generated in rats after immunization with a purified recombinant protein containing the amino acid residues 687-1183 of Frl. The sera were collected with standard methods, and the specificity of the antibody was confirmed by Western blot (Supplementary Figure S1).

### 2.3. Immunohistochemistry

The fixation and immunostaining of *Drosophila* embryos were performed as described in Jankovics and Brunner [8]. The following primary antibodies were used: rabbit anti-Zipper [34] 1:100, rabbit anti-Dia (a kind gift from S. Wasserman, University of California, San Diego, La Jolla, CA, USA) 1:200, rat anti-Frl (described above) 1:500, rabbit anti-DAAM-R4 [30] 1:500, rat anti-FHOS [35] 1:200, chicken anti-GFP (Abcam plc, Cambridge, UK) 1:1000, mouse anti-Flag (Merck KGaA, Darmstadt, Germany) 1:500, mouse 2A12 (Developmental Studies Hybridoma Bank, Iowa City, IA, USA) 1:40. As secondary antibodies, we used the appropriate Alexa-488 or Alexa-546 coupled antibodies (Thermo Fisher Scientific Inc., Waltham, MA, USA) 1:600. Actin was labeled with Alexa-488, Alexa-546, or Alexa-647 coupled phalloidin (ThermoFisher Scientific Inc., Waltham, MA, USA) 1:80. The embryos were mounted in the ProLong Gold antifade reagent (ThermoFisher Scientific Inc., Waltham, MA, USA).

Imaging was performed on a Zeiss LSM880 confocal microscope with an Airyscan detector, using 40x/NA 1.3 oil or 63x/NA 1.4 oil objectives. Images were restored using Huygens Professional (Scientific Volume Imaging B.V., Hilversum, The Netherlands) and Fiji [36] software.

### 2.4. Western Blot Analysis

Western blots were performed by using standard procedures. Rat anti-actin (1:10,000, MAC 237, Abcam), rabbit anti-Dia (1:2000, a gift from S. Wasserman, University of California), rat anti-Frl (1:1000, described above), rabbit anti-DAAM-R4 (1:5000, [30]), and mouse anti-Flag (1:1000, M2, Sigma-Aldrich, St. Louis, MO, USA) were used as primary antibodies. Anti-rabbit-HRP (1:10,000; Jackson ImmunoResearch Europe Ltd., Cambridgeshire, UK) and anti-mouse IgG-HRP (1:5000, Agilent Dako, Santa Clara, CA, USA) were used as secondary antibodies, and proteins were visualized with the chemiluminescent Millipore Immobilon kit.

### 2.5. Live Imaging and Image Analysis

Embryos were dechorionated in 50% bleach, mounted in water onto a glass-bottom cell culture dish (MatTek Corporation, Ashland, MA, USA), and imaged with a Zeiss LSM880 confocal laser scanning microscope using 40x/NA 1.3 oil or 20x/NA 0.8 dry objectives. Every video was acquired at 25 °C. For the analysis of dorsal closure (acquired with a 20x objective), we performed Z-series of 14 planes separated by 1.2 µm and acquired every 4 min (Supplementary Video S1). For AS dynamics (acquired with a 40x objective), we filmed Z-series of 11 planes separated by 0.9 µm and acquired every 30 s (Supplementary Video S2).

All image processing and data analysis were performed using Fiji [36] and Microsoft Excel 2016. 

#### 2.5.1. Trachea Analysis

We determined the number of discontinuous fusion points from the nine anastomosis sites in the dorsal trunk.

#### 2.5.2. AS Cell Dynamics

To measure AS cell contractility, Z-series were maximum-projected and segmented using a Fiji plugin Tissue Analyzer (Supplementary Figure S2, Supplementary Video S3). If required, we manually corrected the segmentation results. Using the wand tool in Fiji, we measured the apical AS cell surface area of the six most central AS cells per embryo in a 15 min time window which began when the dorsal gap was 50 μm wide (Supplementary Figure S2). The selected time window roughly corresponded to the middle of the closure process, and it was suitable for standardization as well as for high quality live imaging. The relative apical area change was normalized as described in Pasakarnis et al. [5]. Using “IF” and “AND” equitation in Excel, we calculated the height and the number of the amplitude from the normalized relative apical area change. 

#### 2.5.3. AS Cell Shape

To measure the regularity of AS cell shape, we divided the actual cell shape area (*AS_area_(t_x_)*) when the dorsal gap was 50 μm wide by the surface of the fitted convex hull (*Ch_area_(t_x_)*) (Supplementary Figure S2), and we continued until the 15 min time window ended. In this way, we obtained a ratio that indicates the convolution level of the segmented cell shape:

(1) $$N\left(t_{x}\right)=AS_{area}\left(t_{x}\right)/Ch_{area}\left(t_{x}\right)$$

The measured AS cells were the same as the ones analyzed for AS cell contractility.

#### 2.5.4. AS Cell Area

We used the wand tool to measure the AS cell area from the segmented Z-series when the dorsal gap was 50 μm wide. The measured AS cells were the same as used for AS cell shape determination.

#### 2.5.5. Filopodia Number and Length

The filopodia number and length were calculated with the line tool within six *en-Gal4* stripes per embryo when the LEs were 30–50 μm apart from each other. 

#### 2.5.6. Dorsal Closure Parameters

The width and height of the dorsal hole were measured on maximum-intensity Z-projections using the line selection tool in Fiji. Timepoint zero was defined right after the germ band was retracted and head involution began. The convergence speed of the LE was measured as described in Pasakarnis et al. [5]. The zipping speed was calculated as follows:

(2) $$v\left(t_{x}\right)=\left((length\left(t_{x}\right)-length\left(t_{x+1}\right)\right)/\Delta t$$

where *v(t_x_)* is the zipping speed, *length(t_x_)* is the length of the dorsal hole in a given frame, *length(t_x+1_)* is the length of the dorsal hole in the following frame, and Δ*t* is the time interval between the frames.

### 2.6. Statistics and Figures

Statistical analysis was carried out using Prism 8 (GraphPad Software Inc., San Diego, CA, USA). The D’Agostino–Pearson omnibus test was used to assess the normality of the data. Significance levels: ns *p* > 0.05, * *p* ≤ 0.05, ** *p* ≤0.01, *** *p* ≤ 0.001, **** *p* ≤ 0.0001. At least three independent experiments were used in the statistical analysis, a summary of the sample numbers and significance levels is shown in Table 1. Figures and drawings were created in Illustrator CS6 (Adobe). 

## 3. Results

### 3.1. Form3, DAAM, Dia, and Frl Localize to AS and Epidermal Cells during Dorsal Closure

**Figure 2:**
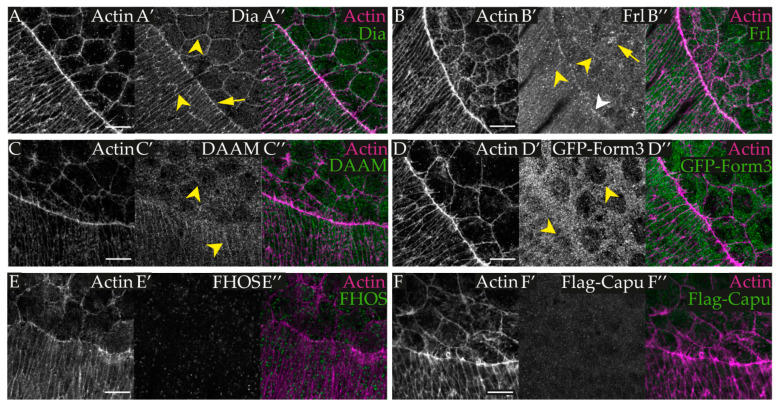
Localization pattern of the formin proteins during dorsal closure. (**A**–**C’’,E**–**E’’**) *w^1118^* embryos, (**D**–**D’’**) *Form3-GFSTF,* and (**F**–**F’’**) *Capu-GFSTF* embryos. Antigens are as indicated. (**A**–**A”**) Dia exhibits a strong cortical localization (arrowheads) both in the LE and AS cells, as well as a junctional accumulation (arrow) in the DME cells. (**B**–**B”**) Frl displays a weak cortical localization (yellow arrowheads) in the DME and AS cells, a weak accumulation along the LE actin cable (white arrowhead), and a non-uniform cytoplasmic accumulation and a mostly punctual nuclear signal in the AS cells (yellow arrow). (**C**–**C”**) DAAM exhibits a cortical localization (arrowheads) in the LE cells and a somewhat weaker one in the AS cells, together with a weak accumulation in the cytoplasm of the AS cells. (**D**–**D”**) The Form3 protein is also present both in the LE and AS cells with a weak cortical and a more profound cytoplasmic accumulation in both cell types (arrowheads). We failed to detect a specific staining pattern in the cases of Fhos (**E**–**E”**) and Capu (**F**–**F”**). Scale bars: 10 μm.

Former work has shown that two diaphanous-related formins (DRFs), Dia and Frl, participate in the regulation of dorsal closure [14,15]. A LOF analysis of *dia^5^* maternal and zygotic mutant embryos revealed defects in epidermal sheet alignment and reduced filopodia numbers, indicating that Dia is primarily required in the LE cells and explaining the slowdown of the closure process [14,28]. Frl is involved in AS cell contractions, and the maternal and zygotic loss of *frl* reinforces the pulsatility of the AS cells. Importantly, however, the lack of Dia or Frl does not prevent the fusion of the epidermis. Given that DC relies on a series of complex cytoskeletal rearrangements in a few different cell types, we assumed that additional actin nucleation factors might also be required, and here we considered the role of all six *Drosophila* formins. To begin this analysis, we performed immunostaining experiments to determine which formins are actually localized to the tissues involved in DC (Figure 1). Consistent with previous reports [14], we detected Dia in the cell cortex of both the epithelial and the AS cells, as well as at the apical adherens junctional region of the DME cells (Figure 2A). Frl is mostly present in the cytoplasm of AS cells and in the cortical region of the epidermal cells (Figure 2B). In addition, a weaker Frl signal was found in the nuclei and cortex of the AS cells, and along the LE actin cable (Figure 2B). Similar to the findings of Flores-Benitez and Knust [37], DAAM showed a significant accumulation in the cortex of the epithelial cells and it was also present in the AS cells (Figure 2C), while Formin 3 exhibited a similar localization pattern as DAAM, although it was stronger in the cytoplasm of the AS cells (Figure 2D). In the cases of Fhos and Capu, we detected only background staining (Figure 2E,F). Thus, the embryonic protein expression pattern of the *Drosophila* formins indicated that four of the six formin proteins are present in the tissues contributing to DC, and the different localization of these four formins may indicate different functions during the process.

### 3.2. Formin LOF Mutations Used during Our Studies

**Figure 3:**
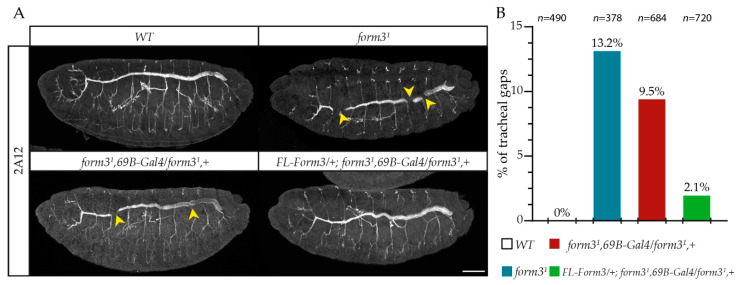
Form3 is required for tracheal development. (**A**) Stage 17 embryos of the genotypes indicated and stained for 2A12 to label the lumen of the tracheal system. Unlike the wild type, *form3^1^* mutant embryos display gaps in their main airways (arrowheads) that can be rescued with the expression of UAS-FL-Form3. Scale bar: 50 μm. (**B**) Quantification of the tracheal gap phenotype of the embryos indicated in panel A. *w^1118^* was used as the wild-type (WT) control.

To investigate the function of these four formins during DC, we initiated a LOF analysis. As controls, we selected *dia^1^*, a hypomorphic allele causing a comparably strong reduction in the Dia level as the formerly used alleles (Supplementary Figure S1) [14], and *frl^59^*, a homozygous viable protein null allele (Supplementary Figure S1) [15]. To explore the function of DAAM, we selected the *DAAM^Ex4^* allele [30]. This is also a homozygous viable allele, strongly reducing the expression of the DAAM-PB isoform (Supplementary Figure S1), which is the major embryonic DAAM isoform until stage 15 of embryogenesis. In the case of *form3*, we used the CRISPR/Cas9 technique to generate a novel null allele, *form3^1^* (Methods), which is a 13.5 kb deletion in *form3* removing 99% of the coding region including the functionally indispensable two formin homology domains (FH1 and FH2). The *form3^1^* allele is semilethal, although homozygous fertile adults hatch and the allele can be maintained as a homozygous stock. Based on previous work, point mutant alleles of *form3* affect the fusion of the main tracheal airways [38]. Thus, to verify our new allele, we investigated the tracheal system in *form3^1^* homozygous embryos. We found that, similar to the findings of Tanaka et al. [38], about 13% of the *form3^1^* embryos exhibit a discontinuous lumen in the dorsal trunk of their tracheal system, which is significantly higher as compared to wild-type controls (Figure 3A,B). We were able to rescue this phenotype by the expression of a wild-type UAS-Form3 transgene in *form3^1^* null mutant embryos using the 69B-Gal4 driver line (Figure 3A,B), and therefore, these results further confirm the role of Form3 in trachea development.

### 3.3. Comparison of the DC Phenotypes of the Formin Mutants

**Figure 4:**
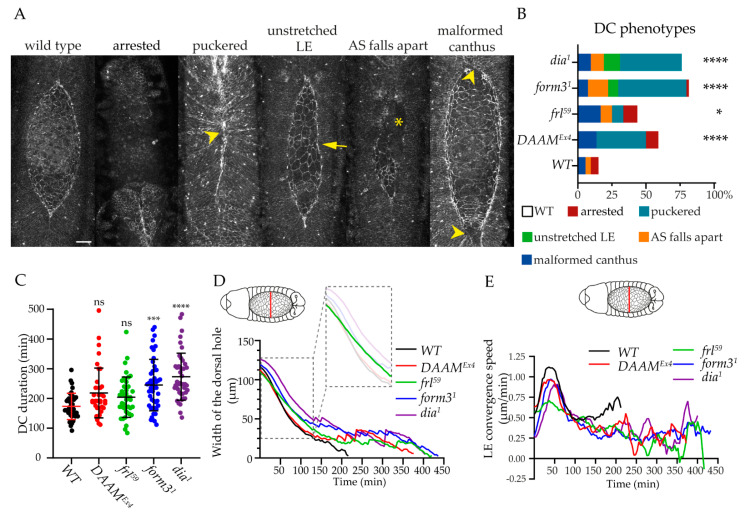
Comparison of the dorsal closure phenotypes in formin mutants. (**A**) Representative images of the dorsal closure phenotypes observed in Arm::GFP-expressing formin mutant embryos. Arrowheads point to the puckered dorsal midline and to the malformed canthi, an arrow indicates the unstretched DME, and an asterisk marks the place of missing AS cells. Scale bar 20 μm. (**B**) Quantification of the morphological defects observed in formin mutant embryos during dorsal closure. The color code represents the previously described categories. *Arm::GFP* was used as the wild-type (WT) control in **B**–**E**, and the mutants were compared to the WT control in **B**–**C**. (**C**) Scatter dot plot of DC duration in the formin mutants. Values are mean ± SD. (**D**–**E**) Diagram of the temporal change in the width of the dorsal hole (**D**) and that of LE convergence speed (**E**) in formin mutant embryos. Values indicate the average of several embryos. The inset in **D** highlights the first phase of the process to illustrate that, in *frl* mutants, the curve is linear, while it is sigmoid in all the other cases. For statistical analysis, we used the Chi-square test (**B**) and the Kruskal–Wallis test *p* < 0.0001, followed by Dunn’s multiple comparisons test (**C**); ns = non-significant. * *p* ≤ 0.05, *** *p* ≤ 0.001, **** *p* ≤ 0.0001.

For the initial assessment of the epithelial closure process in formin mutants, we used live-imaging microscopy where all epidermal cells and AS cells were labeled with Armadillo::GFP. Armadillo is the *Drosophila* homolog of mammalian beta-catenin, a highly conserved adherens junction component. An analysis of the different formin mutant embryos revealed a series of phenotypic effects that we classified into six major groups: wild type, puckered, unstretched LE, AS falls apart, malformed canthus, and arrested DC (due to dying of the embryo). As expected, the majority (about 76%) of the *dia^1^* embryos exhibited an altered gap morphology during DC (Figure 4A,B). The most frequent defect (in about 45% of the embryos) was the puckering of the dorsal epidermis, which is thought to be caused by mismatching of the opposite epithelial sheets [5] (Figure 4A,B). In addition, 12% of the embryos displayed an unstretched LE (Figure 4A,B), 10% had a malformed canthus (Figure 4A,B) meaning that either the anterior or the posterior canthus was missing, and in 10% of the embryos, the AS falls apart (Figure 4A,B). In the case of *frl^59^* mutant embryos, 44% presented a distorted closure where we detected errors in canthus formation (17%), arrested DC (10%), puckering (8%), and the AS falling apart (in 8% of the embryos) (Figure 4A,B). As compared to these, 81% of the *form3^1^* mutant embryos exhibited DC defects that were highly similar to the ones observed in *dia^1^* (Figure 4A,B). In *DAAM^Ex4^*, we revealed DC defects in nearly 60% of the embryos, most of which were puckering (36%) or a failure in canthus formation (14%) (Figure 4A,B). Based on these results, we conclude that all four formins affect DC at this gross morphological level. The effects of *dia* and *form3* are much alike in impairing three aspects of DC: puckering, LE stretching, and AS development. In contrast, *frl* had a stronger effect on canthus formation, and a significant portion of the embryos died before DC was completed, whereas the most profound effects of DAAM were puckering and impaired canthus formation.

Despite the different defects in gap morphology, most of the embryos can still successfully complete DC in the absence of these formins. To begin to analyze the dynamics of the process, we first measured DC duration. In the control embryos, the closure of the dorsal hole was completed within 174 ± 44 min, while in the case of *frl^59^* we could detect an increased time requirement (204 ± 68 min) (Figure 4C), which is comparable to the rate measured in a recent study [15]. In *DAAM^Ex4^,* we also found an increased duration (219 ± 84 min), that was further increased in the *form3^1^* (245 ± 87 min) and *dia^1^* (274 ± 79 min) mutants (Figure 4C). To investigate the duration of the closure further, we measured the convergence of the two opposite epithelial sheets (Figure 4D,E). The decrease in the dorsal hole width followed a sigmoidal curve in the wild-type, *DAAM^Ex4^*, *form3^1^* and *dia^1^* embryos; however, in the *frl^59^* mutants, the curve appeared linear (at least until 150 min) (Figure 4D). In *DAAM^Ex4^* embryos, the width of the hole initially decreased with identical dynamics as in the wild type until 150 min, when the closure was arrested for about 100–120 min (Figure 4D), causing a delay in the completion of the process. In the cases of the other three mutants, the closure curves also revealed a temporal stop between 100 and 140 min, just as in *DAAM^Ex4^* embryos, with the difference that the arrest in closing the gap lasted for nearly 200 min in the *form3^1^*, *dia^1^,* and *frl^59^* mutants (Figure 4D).

In the wild type situation, embryos reach the highest LE convergence speed at ~40 min after the initiation of head involution, and this speed will then gradually decrease in the next 60 min (Figure 4E). The *DAAM^Ex4^* embryos followed this tendency; however, the maximum speed was not as high as in controls (Figure 4E). In *form3^1^* and *dia^1^,* the initial speed of the LE displacement was much lower than in the control (wt: 0.55 µm/min, *form3^1^*: 0.28 µm/min, *dia^1^*: 0.27 µm/min) (Figure 4E), and in *dia^1^* there was a 12 min delay in reaching the maximum speed (Figure 4E). In the case of *frl^59^*, the maximum speed was strongly reduced as compared to the wild type (wt: 1.12 µm/min, *frl^59^*: 0.71 µm/min) (Figure 4E), which might explain the nearly linear change in decreasing the width of the dorsal hole (Figure 4D).

Taken together, these observations suggest that the lack of DAAM had the weakest effect on the duration of the closure process and the convergence of the LE. Similar to the morphological phenotypes, the lack of Form3 and Dia had a very similar effect on these dynamic parameters, with *dia* having a slightly stronger effect in these contexts than *form3*. Curiously, in these formin mutants, the sealing of the dorsal hole temporally halts after 120–150 min when roughly 2/3 or 3/4 of the process is already completed, and the last steps will only be finished after another 150–200 min, which is much slower than in the wild type. We interpret these findings as indications that during the first two hours of DC, the functions of Dia and Form3 are largely dispensable, but they then play a role during the final phases of sealing the epithelium. With regard to Frl, we revealed a considerably different effect on the dynamics of the LE convergence than that of the other three formins. Although Frl had a marginal effect on DC duration, the course of the closure curves exhibited a marked difference both as compared to the wild type and to the other formin mutants, indicating an alteration in the dynamics of the process already from the first steps until the end. Thus, Frl appears to display a differential temporal contribution that is most likely paralleled with a differential spatial requirement as compared to the other three formins.

### 3.4. Form3, DAAM, and Frl Contribute to the Zipping of the Epithelial Sheets

**Figure 5:**
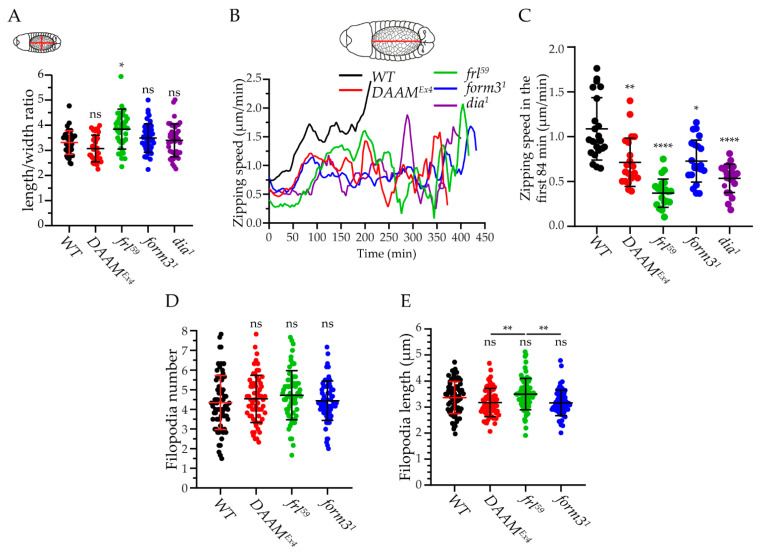
Formin mutants impair zipping of the dorsal hole. *Arm::GFP* was used as the wild-type (WT) control in **A**–**C**, whereas *en-Gal4,UAS-Moe::mCherry* was used as the control in **D** and **E**. (**A**) Scatter dot plot of the length-to-width ratio in formin mutants. Note that only *frl^59^* shows a significant difference as compared to WT, resulting in an elongated dorsal hole. (**B**) Diagram of the temporal changes of zipping speed in formin mutants. (**C**) Diagram of the average zipping speed in the first 84 min of DC (corresponding to the first, very dynamic phase of zipping in WT) in formin mutant embryos. (**D**) Scatter dot plot showing the average filopodia number per engrailed positive stripe in formin mutant embryos. (**E**) Scatter dot plot of the average filopodia length in formin mutants. Statistical analyses were performed by Kruskal–Wallis tests *p* < 0.0001 (**A**,**C**) *p* = 0.0005 (**E**), followed by Dunn’s multiple comparisons test (**A**,**C**,**E**) and by ordinary one-way ANOVA test *p* = 0.3206, followed by Dunnett’s multiple comparisons test (**D**). Mutants were compared to the WT, unless indicated otherwise. Data are presented as mean ± SD; ns = non-significant. * *p* ≤ 0.05, ** *p* ≤0.01, **** *p* ≤ 0.0001.

Besides the changes in DC dynamics, in vivo imaging of the closure process revealed that in some embryos, the dorsal hole was abnormally narrow. To quantify this phenotype, we measured the length-to-width ratio of the hole in all formin mutants at an appropriate timepoint when the width was 30 µm. This ratio was not affected by *dia* or *form3*, while it was significantly increased in the case of *frl^59^* and exhibited a decreasing tendency in DAAM mutants (Figure 5A). Because it is thought that an increased length-width ratio indicates an impairment of the zipping process [39], we next measured the zipping speed in the formin mutant set. In wild-type embryos, the zipping speed linearly increased from 0.7 µm/min up to 1.7 µm/min in about 90 min (Figure 5B), which was followed by a largely steady speed phase for 100 min, to result in a second boost in the speed (up to 2.5 µm/min) during the last 20–30 min of DC (Figure 5B). As compared to this zipping speed curve, the lack of any of the four formins examined here impaired the zippering process. The *DAAM^Ex4^* and *form3^1^* mutants reached their maximum speed during the first phase at the same time as the wild type, however, their maximum was significantly lower (1.2–1.1 µm/min in *DAAM^Ex4^* and *form3^1^* versus 1.7 µm/min in the wild type) (Figure 5B). In *form3^1^,* this speed then decreased below 1.0 µm/min for 250 min and increased again to 1.5 µm/min during the final 30 min (Figure 5B). We observed a rather similar tendency in *DAAM^Ex4^*, with the exception that during the 150 min long decreasing phase, the speed exhibited much higher fluctuations than in *form3* (Figure 5B). In the cases of *dia^1^* and *frl^59^*, the mutants reached their first maximum speed much later than the controls (48 min delay in *dia^1^* and 68 min delay in *frl^59^*) (Figure 5B), suggesting that the first phase of the process was less efficient than in the wild type (Figure 5B). Moreover, the speed at the beginning of the zipping process was significantly lower in these two cases (0.4 µm/min for *frl^59^*, 0.6 µm/min for *dia^1^,* and 0.7 µm/min for the wild type), whereas the average zipping speed during the first 84 min of DC (when the most intensive phase of zipping was taking place) also exhibited strong reductions in the formin mutants (0.7 µm/min for *DAAM^Ex4^*, 0.4 µm/min for *frl^59^*, 0.7 µm/min for *form31*, 0.5 µm/min for *dia^1^,* and 1.1 µm/min for the wild type) (Figure 5C).

The zippering process is mediated by lamellipodia and filopodia-like protrusions produced by the LE cells. As formins have been shown to be required for filopodia formation in several different cell types (reviewed in [40]), and *Drosophila* Dia has been implicated in filopodia formation during DC [28,29], we examined the number and length of the filopodia in the LE cells in the absence of the formins. Surprisingly, by quantifying these parameters, no significant differences were found when compared to wild-type controls (Figure 5D,E), although the filopodia length in *frl^59^* is significantly longer when compared to *DAAM^Ex4^* or *form3^1^* mutants (Figure 5E). The finding that, unlike *dia* [28], these formins are not required for filopodia formation in the LE cells might indicate that their contribution to zipping is independent of filopodia, and implicit to this scenario is that they affect another actin population than the one present in filopodia. An alternative explanation could be an effect on filopodia dynamics instead of filopodia morphology. Finally, it also remains possible that the formin functions are highly redundant with regard to filopodia formation but non-redundant regarding a filopodia-independent aspect of the zipping process.

### 3.5. Formins Differently Regulate the Shape and Contraction Dynamics of AS the Cells

**Figure 6:**
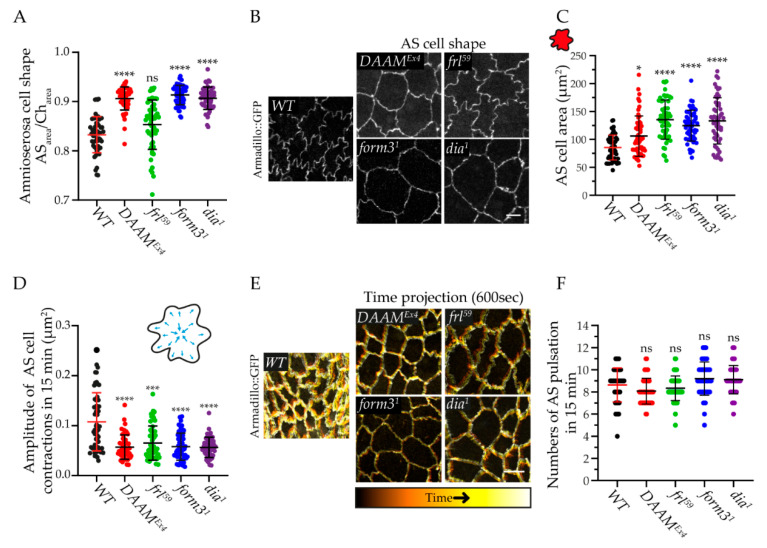
Loss of formins affects the dynamic parameters of the AS cells. *Arm::GFP* was used as wild type (WT) control. (**A**) Scatter dot plot of AS cell shape quantification by determining the ratio of AS area and the area of the fitted convex hull (Ch) in formin mutant embryos. (**B**) Representative images of the AS cell shapes. WT and *frl^59^* embryos show a wavy AS cell border, while the other mutants show a stretched cell border. Scale bar 5 µm. (**C**) Scatter dot plot of the AS cell area in formin mutants when the dorsal hole is 50 µm wide. (**D**) Scatter dot plot of the amplitude of AS cell contractions in formin mutants, measured in a 15 min time window. (**E**) Representative time projections of the AS cell contractions observed in the formin mutants. The width of the line at the cell cortex is proportional to the degree of contractions. Scale bar 10 µm. (**F**) Scatter dot plot of the number of AS cell pulsations during a 15 min time window. Note that the *DAAM^Ex4^* and *frl^59^* embryos show a slightly decreased pulsation activity, while the *form3^1^* and *dia^1^* mutants show a slightly increased pulsation activity. Data sets were analyzed by Kruskal–Wallis tests *p* < 0.0001, followed by Dunn’s multiple comparisons test (**A**,**C**,**D**) and ordinary one-way ANOVA *p* < 0.0001, followed by Dunnett’s multiple comparisons test (**F**). Mutants were compared to the WT. Data are presented as mean ± SD; ns = non-significant. * *p* ≤ 0.05, *** *p* ≤ 0.001, **** *p* ≤ 0.0001.

During the course of our live-imaging studies, we noticed that in some cases the shape of the AS cells looked different than in wild-type. Since it is known that the AS can autonomously drive DC [5,6,41], changes in cell shape and area can be important factors in the regulation of the process. For a quantitative assessment of cell shape, we used a method where a convex hull is fitted on the cells (Methods, Supplementary Figure S2), and we found that, in contrast to the wavy shaped cells in wild-type and *frl^59^* embryos (Figure 6A,B), most AS cells of *DAAM^Ex4^*, *form3^1^,* and *dia^1^* mutants exhibited a more regular, more round shape (Figure 6A,B). In addition, the AS cells appeared significantly bigger in *form3^1^*, *dia^1^,* and *frl^59^* embryos, while in *DAAM^Ex4^* we detected only a slight increase in cell area (Figure 6C). To further investigate this phenomenon, we measured the perimeter of the AS cells, and it was indeed the biggest in *frl^59^* mutants, supporting that the AS cells are big and wavy in this case, while in the case of the other formin mutants, the cell perimeter did not change greatly, indicating that they have bigger and rounder AS cells than those of the wild type (Supplementary Figure S2).

Next, we asked whether the impaired AS cell shape observed in the formin mutants affects the behavior of the AS cells. One of the most striking features of the AS cells is their pulsed contractions, shown to be critically important for DC [5,6]. To quantify the amplitude of the AS cell contractions, we used a 15 min long time window starting at the point of 50 μm gap width (Methods). A decrease in the amplitude of the cell contraction is obvious in the case of all four formin mutants examined (Figure 6D), and this effect is well visible in color-coded time projection images where the width of the line at the cell cortex is proportional to the degree of contractions (Figure 6E). The level of reduction was very similar in all mutants, falling into the range of 50–60%. We also measured the pulsation frequency in the same time window, and we found that, in *DAAM^Ex4^* and *frl^59^* embryos, the number of pulsations tends to somewhat decrease as compared to the wild type (Figure 6F), while the pulsation frequency is slightly increased in the cases of the *form3^1^* and *dia^1^* mutants (Figure 6F)—although these tendencies do not appear to represent statistically significant changes. Collectively, these observations indicate that each formin is required for the efficient contraction of the AS cells, but again, as implied by the opposite effect on the pulsation frequency, DAAM and Frl might have a differential contribution as compared to Form3 and Dia. Interestingly, in *form3* and *dia* mutants, the changes in AS cell shape are also very similar to each other; however, the AS cell shape in *DAAM* and *frl* mutants differ from one another, suggesting that AS cell shape alone is not an indicator of the dynamics of the contractions.

It has been reported that the overexpression of the constitutively active form of Dia in the AS increases myosin levels [14]. Likewise, the overexpression of Frl causes an increased medial F-actin level in AS cells [15], while in the absence of *frl,* the density of this actin network is decreased [15]. Therefore, to examine how formins affect the acto-myosin system of the AS cells, we performed immunostaining in all four mutants. By investigating the actin and myosin levels, we found a slight decrease in AS cell actin density in all mutants, but we did not find a convincing change in myosin density (Supplementary Figure S3), and thus, these data are consistent with formins primarily being involved in actin regulation.

## 4. Discussion

Mounting evidence suggests that DC is driven by the combination of multiple forces generated by amnioserosa contraction, actin cable tension, and the zipping of the epidermal cells (reviewed in [1,42,43,44,45]). As expected, F-actin, actomyosin, and microtubule-based cytoskeletal mechanisms play a pivotal role in this sealing process. While the contribution of branched actin networks has not been reported, linear actin cables are required for the pulsatile contraction of AS cells, LE actomyosin cable formation, and LE cell protrusions. Consistent with the notion that the bulk of unbranched actin filaments are assembled by formin proteins, two *Drosophila* formins (Dia and Frl) have already been shown to be involved in DC. Specifically, Dia is required for filopodia formation in the LE and AS cells [14,28,29], while Frl is implicated in the formation of a persistent, medioapical actin subpopulation in the AS cells promoting the propagation of Myosin-II-induced contractile forces [15]. These observations suggest that Dia and Frl are required in two different cell types, and consequently, they regulate at least two different actin populations. Importantly, it has also been shown that these two formins are not essential for sealing the dorsal hole; it is mainly the cellular dynamics of the process that are impaired in *dia* and *frl* mutant embryos. To clarify whether additional formins are involved, we initiated a comparative analysis of all six *Drosophila* formins. These studies established that, in addition to Dia and Frl, Form3 and DAAM are also expressed in the LE and AS cells during DC, and in harmony with this, the *form3* and *DAAM* mutants exhibit morphological alterations during DC. Thus, the closing of the dorsal epidermis requires the concerted action of four formins, each displaying a different phenotype when analyzed in detail.

The comparison of the embryonic expression pattern of the six *Drosophila* formins revealed that Dia, Frl, Form3, and DAAM are present in the lateral epidermal cells as well as in the AS cells during DC, while Fhos and Capu do not exhibit a specific accumulation in these regions. Despite being present, it is notable that the localization patterns of Dia, Frl, Form3, and DAAM are different from one another. Dia strongly accumulates at the cortex of both the LE and AS cells, which is similar to the localization of DAAM, although DAAM staining is less strong along the LE actin cable, it is more punctual along the cortex of the AS cells, and the cytoplasmic signal in the AS cells is stronger than for Dia. As compared to this, Frl and Form3 exhibit a relatively weak cortical accumulation in the epidermal cells, and a stronger, mostly cytoplasmic staining in the AS cells. Importantly, while Frl mainly accumulates into cytoplasmic foci in the medial region of the cells, the Form3 pattern is more uniform in a wide cytoplasmic zone along the cortex of the AS cells. The medioapical and mediolateral Frl enrichments are consistent with a role in the assembly of a persistent, medioapical actin network, as proposed recently [15]. In addition, the punctate nature of the cytoplasmic Frl accumulations indicates that the Frl-dependent actin cables emanate from spatially controlled foci.

In accordance with the protein localization data, the genetic impairment of the four formins expressed in the LE and AS cells caused various phenotypic defects during DC, including morphological alterations and changes in the dynamic parameters of the process. Gross morphological defects were most frequent (~80%) in the *form3* and *dia* mutant embryos, while the penetrance of the DC phenotypes is 60% in the *DAAM* and 44% in the *frl* mutants. In the cases of *form3*, *dia,* and *DAAM,* the majority of the defects comprised a puckering of the dorsal epidermis, although *form3* and *dia* also affected LE stretching and AS development, and DAAM impaired canthus formation. In the absence of Frl, canthus formation was often compromised and a significant number of the mutant embryos died before DC was fully completed. Although we found that most embryos can still close the dorsal hole despite these diverse morphological defects, we revealed that the DC dynamics are strongly affected by the lack of the formins. To provide a more complete picture, we measured a set of dynamic parameters in each formin mutant to follow the convergence of the LE, the zipping process, and the contraction of the AS cells. These studies revealed that the DC duration was longer in the formin mutants, in particular in the cases of *form3* and *dia*, and the speed of LE convergence was reduced in all mutants. Moreover, in *DAAM*, *form3,* and *dia* mutant embryos, the convergence of the LE was largely normal during the first two hours of the process when a temporal stop appeared to slow down the sealing of the gap. In contrast, in the absence of Frl, the closure curve shows an alteration in the closing process from the beginning. Thus, these data indicate that the effects of *form3*, *dia,* and *DAAM* are similar to each other, both with regard to the morphological phenotypes and to the DC dynamics, whereas *frl* has distinct effects in both respects. These differences are best explained by different temporal, and presumably, spatial requirements for the two classes of formins, i.e., Frl being required from the early phases of DC, while Form3, Dia, and DAAM provide an important contribution only during the final phases. This hypothesis is in line with the formerly demonstrated role of Frl in AS cell contraction, which is thought to be a major force-generating process from the onset of DC [15], and with the finding that Dia plays a role in zipping [14,28] which is thought to be more important in the second half of DC. Curiously, however, we found that zipping was slowed down in all formin mutants, including *frl*, and conversely, AS cell dynamics were not only affected by *frl*, but also by the other three formins. As to zipping, unlike Dia, Form3, DAAM, and Frl are not required for filopodia formation, and unless they regulate filopodia dynamics, they may contribute to zipping by regulating a non-filopodial actin subpopulation. One candidate for this would be the supracellular actomyosin cable at the LE/AS boundary, which is however not significantly disrupted in the formin mutants, and therefore it might also be an as yet unidentified F-actin population. Regarding the behavior of the AS cells, our results revealed that each formin is required for the efficient contraction of these cells. Nevertheless, where *DAAM* and *frl* reduce, *form3* and *dia* tend to slightly increase the frequency of AS cell pulsation, possibly indicating a differential contribution. Collectively, the analysis of DC dynamics in the formin mutants strongly suggests that each of the four formins is involved in zipping and AS cell contraction as well. It is striking that, despite their role in AS contractions, genetic perturbation of the formins does not strongly affect the first phase of DC but mostly impacts the final stages of the process. To explain this finding, we propose that the first phase is much less dependent on the presence of formin specific actin subpopulations, whereas formin specific cytoskeletal elements are required for efficient completion of the final stages. Alternatively, because formins are also implicated in mechanotransduction [46], it is also conceivable that proper force coordination during the final stages involves the concerted action of several formins, each with a specific role, while the first half of DC is less dependent on non-redundant force-sensing mechanisms. We note that such a role might also explain the AS cell shape changes observed upon *dia*, *form3,* and *DAAM* depletion.

Another important conclusion of the comparative study of DC dynamics is that the different formins play different roles in the process, implying that each formin regulates the assembly of distinct actin bundles in the DME and AS cells. Even so, it is noteworthy that the two formins exhibiting the most similar phenotypic effects, *dia* and *form3*, belong to two separate formin subfamilies. Dia, together with DAAM and Frl, is a member of the DRF subfamily that is regulated by a well-characterized intramolecular autoinhibitory mechanism, whereas Form3 is the sole *Drosophila* inverted formin, the activity of which is regulated by an unknown mechanism. The activity of the DRF subfamily is regulated by Rho family small GTPases (Rho, Rac, and Cdc42), all of which have already been linked to DC [14,47,48,49,50], making them good candidates for Dia, DAAM, and Frl activation during DC. Indeed, Dia is likely to be regulated by Rho1 [14], but the mechanism of Frl and DAAM activation in this context remains less clear [15]. Independent of their precise modes of activation, the phenotypic similarities between *dia* and *form3* indicate that, with the exception of filopodial actin, they may contribute to the formation of an at least partly overlapping set of actin populations, which are distinct from the Frl and DAAM dependent actin networks. Thus, we propose that the four formins promote the formation of numerous, and only partly identical, actin structures, each with a specific role in DC mechanics. By considering that DC requires the orchestration of several different forces in several different cell types, the involvement of at least four actin assembly factors is entirely conceivable.

In summary, we have shown here that four DC-related formins differentially regulate the dynamics of embryonic dorsal closure. Despite playing significant roles, none of these formins by themselves were essential to the success of the process, further confirming that this key morphogenetic event is highly robust and secured by multiple cytoskeletal mechanisms. The known molecular function of the formin protein family suggests that Dia, Form3, DAAM, and Frl are required for the assembly of various F-actin subpopulations critical to proper force generation in the LE and AS cells during DC. Thus far, only one such Frl-dependent actin subpopulation has been identified in AS cells, and although LE filopodia formation is known to be Dia-dependent, the identification of the additional formin-dependent actin structures awaits future elucidation.

## Figures and Tables

**Table 1:** Summary table of the sample numbers used and the significance levels obtained during analysis of the effects of the formin mutants on the different aspects of the dorsal closure process. All mutant embryos were compared to the WT control. n.d. = not done, ns= non-significant.

		*WT*	*DAAM^Ex4^*	*frl^59^*	*form3^1^*	*dia^1^*
Dorsal closure	Closure phenotype	*n* = 53 embryos	*p* < 0.0001 *n* = 44	*p* = 0.0196 *n* = 48	*p* < 0.0001 *n* = 54	*p* < 0.0001 *n* = 42
	DC duration	*n* = 38 embryos	ns *n* = 37	ns *n* = 40	*p* = 0.0002 *n* = 46	*p* < 0.0001 *n* = 44
	LE convergence speed	*n* = 23 embryos	ns *n* = 17	*p* = 0.0019 *n* = 14	ns *n* = 17	*p* = 0.0247 *n* = 8
	zipping speed	*n* = 23 embryos	*p* = 0.0075 *n* = 17	*p* < 0.0001 *n* = 14	*p* = 0.0065 *n* = 17	*p* < 0.0001 *n* = 8
	length/width ratio	*n* = 40 embryos	ns *n* = 39	*p* = 0.0104 *n* = 39	ns *n* = 47	ns *n* = 42
Amnioserosa	AS cell area	*n* = 54 cells	*p* = 0.0387 *n* = 54	*p* < 0.0001 *n* = 54	*p* < 0.0001 *n* = 54	*p* < 0.0001 *n* = 54
	AS cell perimeter	*n* = 54 cells	ns *n* = 54	*p* < 0.0001 *n* = 54	ns *n* = 54	*p* = 0.018 *n* = 54
	AS cell contraction	*n* = 54 cells	*p* < 0.0001 *n* = 54	*p* = 0.0002 *n* = 54	*p* < 0.0001 *n* = 54	*p* < 0.0001 *n* = 54
	AS cell shape	*n* = 54 cells	*p* < 0.0001 *n* = 54	ns *n* = 54	*p* < 0.0001 *n* = 54	*p* < 0.0001 *n* = 54
	AS cell pulsation	*n* = 54 cells	ns *n* = 54	ns *n* = 54	ns *n* = 54	ns *n* = 54
Filopodia	filopodia number	*n* = 72 embryos	ns *n* = 64	ns *n* = 75	ns *n* = 64	n.d.
	filopodia length	*n* = 69 embryos	ns *n* = 64	ns *n* = 75	ns *n* = 64	n.d.

## Data Availability

The datasets generated and/or analyzed during the current study are available from the corresponding author upon request.

## Supplementary Materials

The following supporting information can be downloaded at: https://www.mdpi.com/article/10.3390/cells11091539/s1, Figure S1: Western blot analysis of stage 13 embryos; Figure S2: Quantification of the AS cell perimeter in formin mutant embryos; Figure S3: The comparison of actin and non-muscle MyosinII distribution in the AS cells of formin mutant embryos; Video S1: Dorsal closure in a wild-type (*Arm::GFP* expressing) embryo; Video S2: AS cell movements in a wild-type (*Arm::GFP* expressing) embryo; Video: S3: AS cell segmentation in a wild-type (*Arm::GFP* expressing) embryo.
