# Supplementary material for: A Comparative Study of the Role of Formins in Drosophila Embryonic Dorsal Closure
Source: Cells. 2022 May 4;11(9):1539. doi: 10.3390/cells11091539 (PMC9102720; doi:10.3390/cells11091539)
Supplement: Supplementary file 1 [file cells-11-01539-s001.zip › SupplementaryMaterials_Toth.pdf]

Supplementary Materials

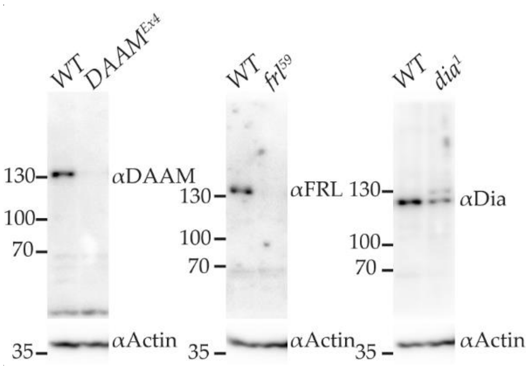

Figure S1. Western blot analysis of stage 13 embryos. Genotypes and antigens as indicated. Note the strongly reduced DAAM protein level in *DAAM^{Ex4}*, the complete absence of Frl in *frl^{59}* and the reduced Dia level in *dia^{1}* mutant embryos. Actin is used as loading control.

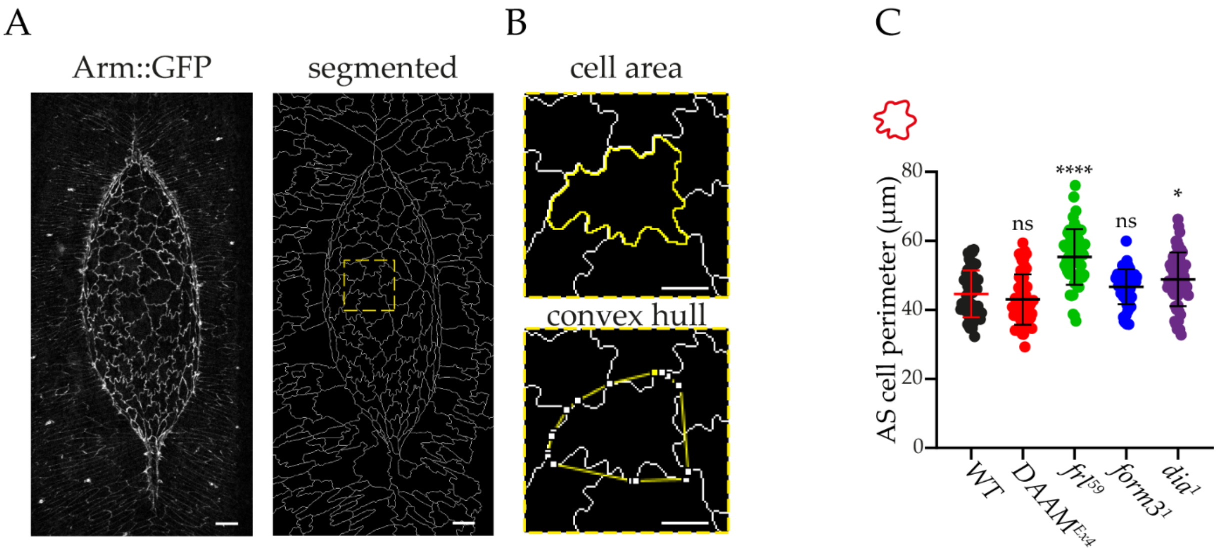

Figure S2. Quantification of the AS cell perimeter in formin mutant embryos. (**A**) Z-projection of time-lapse images from an Arm::GFP expressing embryo when the dorsal hole is 50 µm wide, and a segmentation of the Z-projection. Scale bar 10 µm. (**B**) An example for the segmented AS cell boundary and the fitted convex hull (both in yellow). Scale bar 5 µm. (**C**) Scatter dot plot of the AS cell perimeter in formin mutant embryos when width of the dorsal hole is 50 µm. Values are mean ± SD. Data sets were analyzed by Ordinary one-way ANOVA P<0.0001, followed by Dunnett's multiple comparisons test. Mutants were compared to the WT; ns= non-significant.

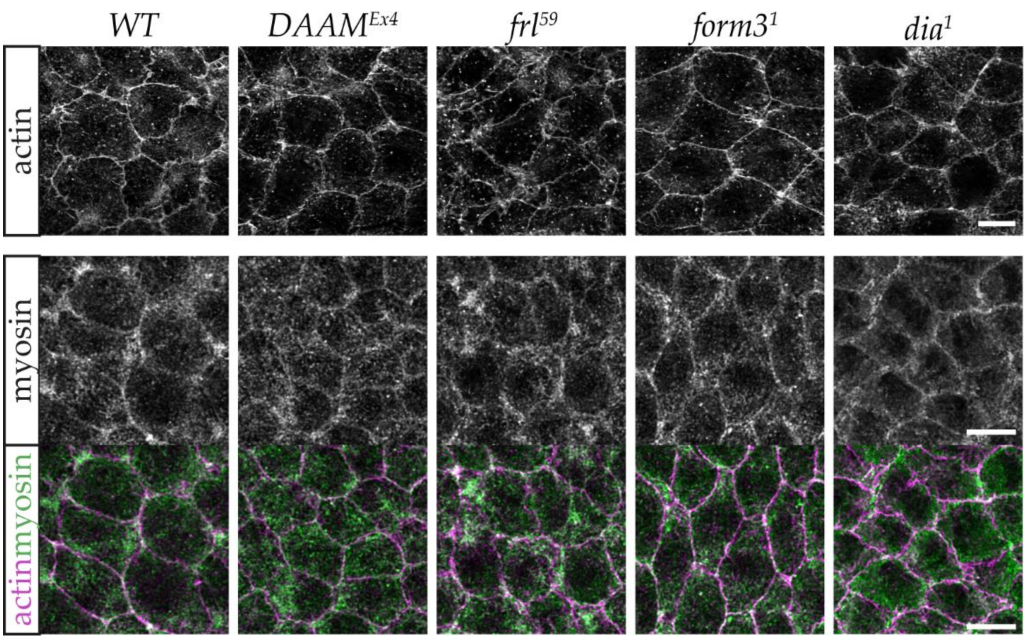

Figure S3. The comparison of actin and non-muscle MyosinII distribution in the AS cells of formin mutant embryos. Apical region of the AS cells is shown from stage 14 embryos of the genotypes indicated, stained for actin and non-muscle MyosinII. In wild type, both actin and MyosinII exhibit a cortical enrichment and a largely diffuse cytoplasmic staining. In formin mutants the cytoplasmic actin signal appears to be slightly reduced as compared to wild type. The MyosinII pattern in the formin mutants appears largely similar to that of wild type. Scale bars 10 µm.

Description of the Supplementary Videos included:

Video S1. Dorsal closure in a wild type (*Arm::GFP* expressing) embryo. Live ×20 imaging. The video represents a maximum projection of 14 Z-planes, separated by 1.2 µm and acquired in every 4 minutes.

Video S2. AS cell movements in a wild type (*Arm::GFP* expressing) embryo. Live ×40 imaging. The video represents a maximum projection of 11 Z-planes, separated by 0.9 µm and acquired in every 30 seconds.

Video S3. AS cell segmentation in a wild type (*Arm::GFP* expressing) embryo. Left: the original video, right: the video after segmentation. Live ×40 imaging. The video represents a maximum projection of 11 Z-planes, separated by 0.9 µm and acquired in every 30 seconds.
